# A Five-Gene Signature Associated With DNA Damage Repair Molecular Subtype Predict Overall Survival for Hepatocellular Carcinoma

**DOI:** 10.3389/fgene.2022.771819

**Published:** 2022-01-20

**Authors:** Junyu Huo, Xinyi Fan, Bingxin Qi, Peng Sun

**Affiliations:** ^1^ Liver Disease Center, The Affiliated Hospital of Qingdao University, Qingdao, China; ^2^ Department of Allergy, The Affiliated Hospital of Qingdao University, Qingdao, China; ^3^ School of Public Health, Qingdao University, Qingdao, China; ^4^ Department of Hepatobilary and Pancreatic Surgery, The Affiliated Hospital of Qingdao University, Qingdao, China

**Keywords:** hepatocellular carcinoma, DNA damage repair, prognostic, signature, risk score

## Abstract

**Background:** DNA damage repair (DDR) is an important mechanism for the occurrence and development of hepatocellular carcinoma (HCC), but its impact on prognosis has not been fully understood.

**Materials and methods:** A total of 904 HCC patients were included in our study, TCGA (*n* = 370) and GSE14520 (*n* = 239) were merged into a large-sample training cohort (*n* = 609). The training cohort was clustered into C1 and C2 based on prognostic DDR-related genes, the differentially expressed genes (DEGs) between C1 and C2 were identified by the Wilcoxon signed-rank test referred to criteria (|log2FC|≥1 and FDR< 0.05). The univariate Cox analysis was used to screen the prognostic-related DEGs, and Lasso penalized Cox regression analysis was used to construct the risk score. The patients were clarified into high- and low-risk groups based on the median risk score. ICGC (*n* = 231) and GSE116174 (*n* = 64) cohorts were used for external validation of the risk score’s prognostic value.

**Results:** The Kaplan–Meier survival analysis showed that the high-risk group had a significantly reduced overall survival (OS) compared to the low-risk group in the three independent cohorts, and the time-dependent ROC curve showed that the five-gene (STMN1, PON1, PLOD2, MARCKSL1, and SPP1) risk score with a high accuracy in predicting OS. The patients with AFP >300 ng/ml, tumor poor differentiation (grade 3–4), micro and macro vascular tumor invasion, advanced stage (AJCC III-IV, BCLC stage B-C, and CLIP score >2) exhibited a higher risk score. Subgroup survival analysis found that the risk score was applicable to patients with different clinical characteristics. GO and KEGG functional enrichment analysis revealed that cell cycle, p53 signaling, TNF signaling-related pathways were upregulated in the high-risk group. The higher infiltration level of activated CD4 T cell, CD56 bright natural killer cell, plasmacytoid dendritic cell, and type 2 T helper cells were found to lead an unfavorable impact on the OS of HCC patients, and these four kinds of immune cells exhibited a higher infiltration level in the high-risk group.

**Conclusion:** The five-gene risk score proposed in the research may provide new insights into the individualized evaluation of HCC prognosis.

## Background

Somatic cells are often affected by a variety of *in vivo* and *in vitro* factors, such as ionizing radiation (IR), ultraviolet (UV) and other physical factors (Soutoglou and Misteli, 2008), alkylating agents, nitrosamines and other chemical factors (Gidron et al., 2006), virus infection and other biological factors (Lewis et al., 2013), these factors will cause a variety of DNA damage, such as single-strand and double-strand gaps (SSB, DSB), base mutation, base oxidative damage and so on (Jackson and Bartek, 2009). If the damage was repaired correctly and the cells survive, and if there were incorrect repairs such as deletions and insertions in the repair process, the accumulation of these abnormal bases may lead to cancer (Alhmoud et al., 2020). If the damage was serious and could not be repaired, the cell would initiate the apoptosis process and induce cell death, which was also considered to be the last barrier to prevent the malignant transformation of the cell (Roos and Kaina, 2006). Therefore, signal transduction, damage repair, and apoptosis induction form a complex network which was closely related and influence each other, which enables cells as a whole to respond to DNA damage.

Hepatocellular carcinoma (HCC) is a primary liver cancer derived from hepatocytes, accounting for 85∼90% of all primary liver cancers (Ozakyol, 2017). It is the fifth most common cancer in men and the seventh most common cancer in women worldwide; it is estimated that there are about 782,000 new cases every year, causing 600,000 deaths every year (Ozakyol, 2017). Its high mortality and short survival time lead to a serious global health burden (Sayiner et al., 2019). The occurrence of HCC is a complex process of interaction between genetic and environmental factors (Aravalli et al., 2008), and its mechanism has not been fully elucidated. Important risk factors include environmental toxins such as chronic viral hepatitis, liver cirrhosis, and aflatoxin, lifestyle factors such as non-alcoholic fatty liver disease (NAFLD), drinking, smoking, and diet (Gomaa et al., 2008). Various HCC related risk factors could cause DNA damage (Schütte et al., 2009). If the damaged DNA was not repaired in time and correctly, it could lead to gene mutation and genomic instability, which is gradually considered to be the common feature of human HCC (Farazi and DePinho, 2006). The disorder of the DNA damage repair process was related to the susceptibility to liver cancer, and this process was often enhanced in HCC cells, resulting in the unsatisfactory effect of anticancer treatment against HCC cells (Yang et al., 2014).

DNA damage repair (DDR) is not only an important mechanism for the occurrence and development of HCC but also an important reason for the poor effect of chemotherapy and other treatments. However, the carcinogenic mechanism of DDR in HCC remains to be investigated, and the improvement and innovation of individualized evaluation of HCC prognosis have a broad prospect, which is worthy of further exploration.

## Materials and Methods

### Data Acquisition

Four independent HCC cohorts with prognosis information were included in our research: TCGA-LIHC, *n* = 370; GSE14520, *n* = 239; ICGC-LIRI-JP, *n* = 231; GSE116174, *n* = 64. Their gene expression and clinical data were obtained from three public databases: The Cancer Genome Atlas (TCGA, https://portal.gdc.cancer.gov/), the International Cancer Genomics Consortium (ICGC, https://icgc.org/), and Gene Expression Omnibus (GEO, https://www.ncbi.nlm.nih.gov/geo/). The clinicopathological information for all cohorts is shown in Table 1. The TCGA and GSE14520 were merged into a large-sample training cohort (*n* = 609). The usage rules of the TCGA, ICGC, and GEO database were fully complied with during data collection. The gene expression format in the three RNA-seq cohorts were normalized to transcripts per million kilobase (TPM) values based on R package “limma”, and the ComBat function of the R “SVA” package was used to eliminate the batch effect in different datasets (Huo et al., 2021e; Huo et al., 2021f). As the data utilized in our research were acquired from public databases, approval from the local ethics committee was not needed.

**TABLE 1:** The clinicopathological information for all cohorts.

	TCGA	GSE14520	ICGC	GSE116174
Survival status				
alive	240	143	189	37
dead	130	96	42	27
Gender				
male	249	189	170	6
female	121	28	61	58
Age				
≤65	232	198	89	55
>65	138	19	142	9
HBV				
Positive				47
Negative				17
Alcohol				
Yes				13
None				51
Grade				
G1	55			
G2	177			
G3	121			
G4	12			
AJCC TNM stage				
I&II	256	168	141	53
III&IV	90	49	90	11
priorMalignancy				
None			201	
Yes			30	
AFP				
≤300 ng/ml	197	120		
>300 ng/ml	62	97		
vascular_tumor_cell_type				
None	206			35
Micro and macro	108			29
new_tumor_event_after_initial_treatment				
None	162			
Yes	168			
BCLC stage				
0-A		165		
B-C		52		
CLIP_Score				
<2		169		
≥2		48		
ALT				
≤50U/L		127		
>50U/L		90		

### DNA Damage Repair Related Genes Cluster Analysis

We extracted 223 DNA damage repair (DDR) pathways (including homologous recombination (HR), mismatch repair (MMR), base excision repair (BER), nucleotide excision repair (NER), and nonho-mologous end-joining (NHEJ) related genes from the molecular signatures database (MSigDB, http://www.gsea-msigdb.org/gsea). Based on the prognostic related genes (PRGs) with *p*-value < 0.05 screened out by the univariate Cox regression analysis, the training cohort were conducted cluster analysis by R-package “ConsensusClusterPlus”. The Kaplan-Meier method with a two-sided log-rank test was employed to compare the overall survival (OS) difference between different clusters. The gene sets enrichment analysis (GSEA) was applied to assess the DDR pathway activities for different clusters. The differentially expressed genes (DEGs) between different clusters were identified by R package “limma” referred to criteria (|log2FC|≥1 and FDR <0.05) (Huo et al., 2021d).

### Development and Validation of a Risks Score Predicting OS of HCC

The DEGs with *p*-value < 0.001 obtained from the univariate Cox regression analysis were considered to be the prognostic related genes (PRGs) in the training cohort (*n* = 609). Next, least absolute shrinkage and selection operator (LASSO) regression with 10-fold cross-validation was performed, and 1,000 cycles were run via the R software package “glmnet”. For each cycle, random stimulation was set to 1,000 times, and the penalty parameter (λ) was decided by the minimum partial likelihood deviance (Huo et al., 2021c). The genes with nonzero regression coefficients obtained from lasso regression analysis were included in the multivariate Cox regression analysis (Huo et al., 2021h). The risk score was established by the expression level of each gene multiple its corresponding regression coefficients derived from multivariate Cox regression analysis of each gene (Huo et al., 2021g). After each patient in the training cohort (*n* = 609) got their own risk score, we arranged them in a sequence from low to high, and took the median value to divide them into a high-risk group and low-risk group (Huo et al., 2021a; Huo et al., 2021b), the Kaplan–Meier survival analysis was implemented to compare the OS of the two groups. The time-dependent receiver operating characteristic (ROC) analysis was used to evaluate the accuracy of the risk score in predicting OS of HCC. The univariate and multivariate Cox regression analysis were used to assess the independent prognostic value of the risk score. Internal validation was conducted in TCGA and GSE14520 cohorts, external validation was conducted in ICGC and GSE116174 cohorts, clinical subgrouping validation was used to test the risk score’s universal applicability.

### Exploration of the Molecular Mechanism Underlying the Prognostic Signature

The DEGs between the high- and low-risk groups were identified by the “limma” R package (fdr <0.05), the R package “clusterProfiler” was employed for the Gene Ontology (GO) and Kyoto Encyclopedia of Genes and Genomes (KEGG) functional enrichment analysis of the DEGs.

### Quantification of 23 Types of Immune Cells Infiltration Using ssGSEA Algorithm

The single sample gene set enrichment analysis (ssGSEA) algorithm was employed to calculate the normalized enrichment score (NES) for the quantification of 23 types of immune cells infiltration abundance (Barbie et al., 2009). The immune cells infiltration differences between the high- and low-risk groups were compared by the independent-sample t-tests, *p* < 0.05 was considered to be of statistical significance.

## Results

### Identification of DEGs Between Different DDR Clusters

**FIGURE 1:**
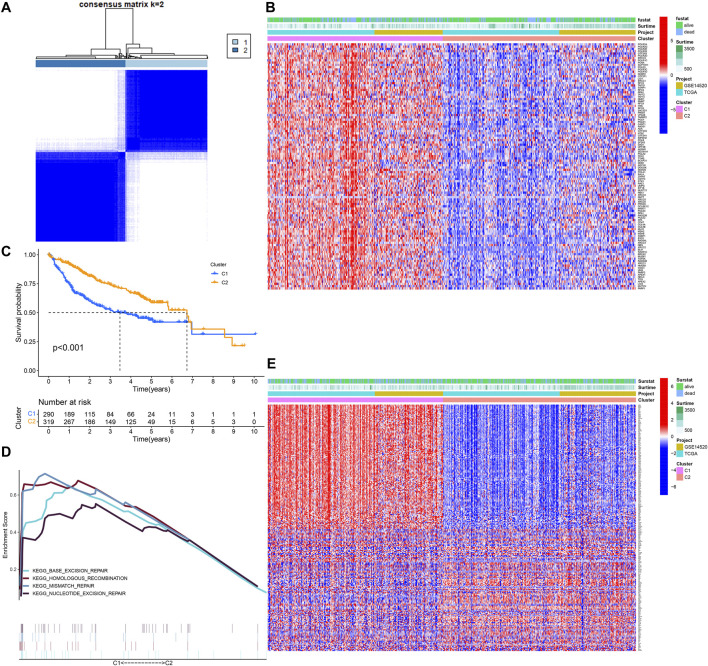
Identification of DNA damage repair related molecular subtype. **(A)** DNA damage repair-related genes cluster analysis. **(B)** The heatmap of DDR clusters. **(C)** The Kaplan–Meier survival analysis. **(D)** Gene sets enrichment analysis. **(E)** The heatmap of DEGs between C1 and C2.

We conducted the univariate Cox regression analysis on the DDR-related genes and found that 108 genes were associated with the OS of HCC (Supplementary Material-1). The training cohort was clustered into C1 and C2 based on more than 108 genes (Figures 1A,B). The OS of C1 was obviously lower than that of C2 (Figure 1C), and the GSEA showed that the C1 was an active DDR activity enhanced subtype (Figure 1D). A total of 239 DEGs were identified between C1 and C2, 166 were upregulated in C1 and 74 were upregulated in C2 (Figure 1E). The Chi-square test showed that there were significant differences between the two subtypes in survival status (*p* = 0.002), but there was no difference between TCGA and GSE14520 (*p* = 0.959) (Supplementary Figure S1).

### A Five-Gene Risk Score Constructed in the Training Cohort

**FIGURE 2:**
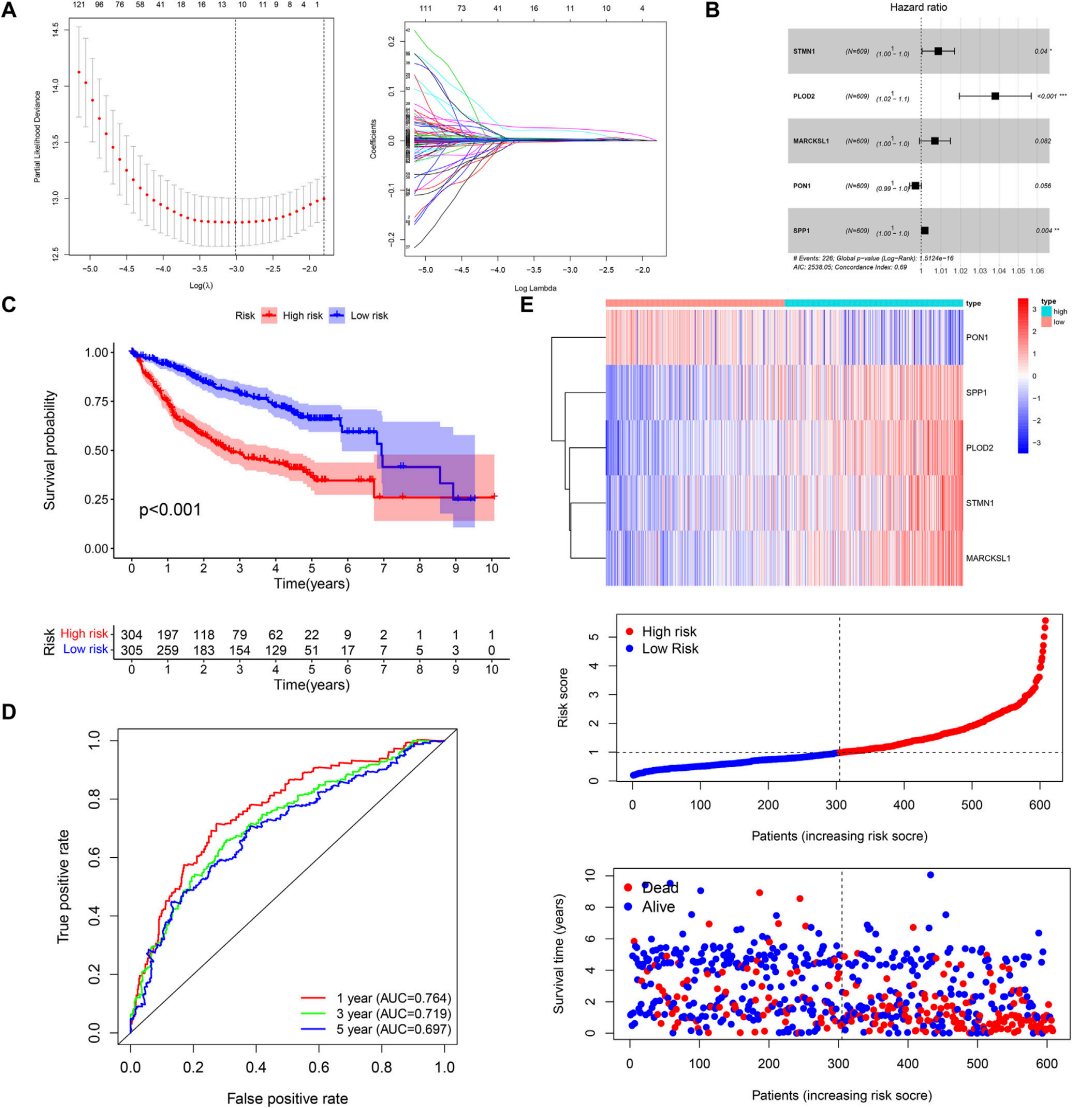
Construction of the five-gene risk score. **(A,B)** LASSO and multivariate Cox regression analysis. **(C,D)** The Kaplan–Meier survival and time-dependent ROC curves. **(E)** The heatmap, risk score distribution, and survival status of patients in the training cohort.

In all, 193 of 239 DEGs were considered to have a significant impact on the prognosis of HCC via univariate Cox regression analysis (*p* < 0.001) (Supplementary Material-2). Through the penalty parameter (λ) was decided by the minimum partial likelihood deviance, 11 genes with nonzero lasso regression coefficient were retained (Figure 2A), a five-gene risk score were formatted by gene expression level and corresponding multivariate Cox regression coefficients: *STMN1* * 0.008619 − *PON1* * 0.002848 + *PLOD2* * 0.03729 + *MARCKSL1* * 0.006922 + *SPP1* * 0.001817 (Figure 2B). The patients were divided into high- and low-risk groups referred to the median risk score (0.986). The OS of the high-risk patients was significantly reduced compared to the low-risk patients (*p* < 0.001, Figure 2C). The area under curve (AUC) values of 1-year, 3-year, and 5-year OS predicted by risk score were 0.764, 0.719, and 0.697 respectively (Figure 2D). The patients with lower risk scores were found to have a longer survival time and higher survival rate (Figure 2E). By comparing AUC, the 1, 3, and 5 years OS prediction efficiency of the risk score was better than that of single gene and clinical factors (Supplementary Figure S2). We also detected the expression of signature genes in human normal tissues and HCC tissues with the help of HPA database (Human Protein Atlas, https://www.proteinatlas.org/) (Supplementary Figure S3).

### Internal and External Validation of the Prognostic Signature

**FIGURE 3:**
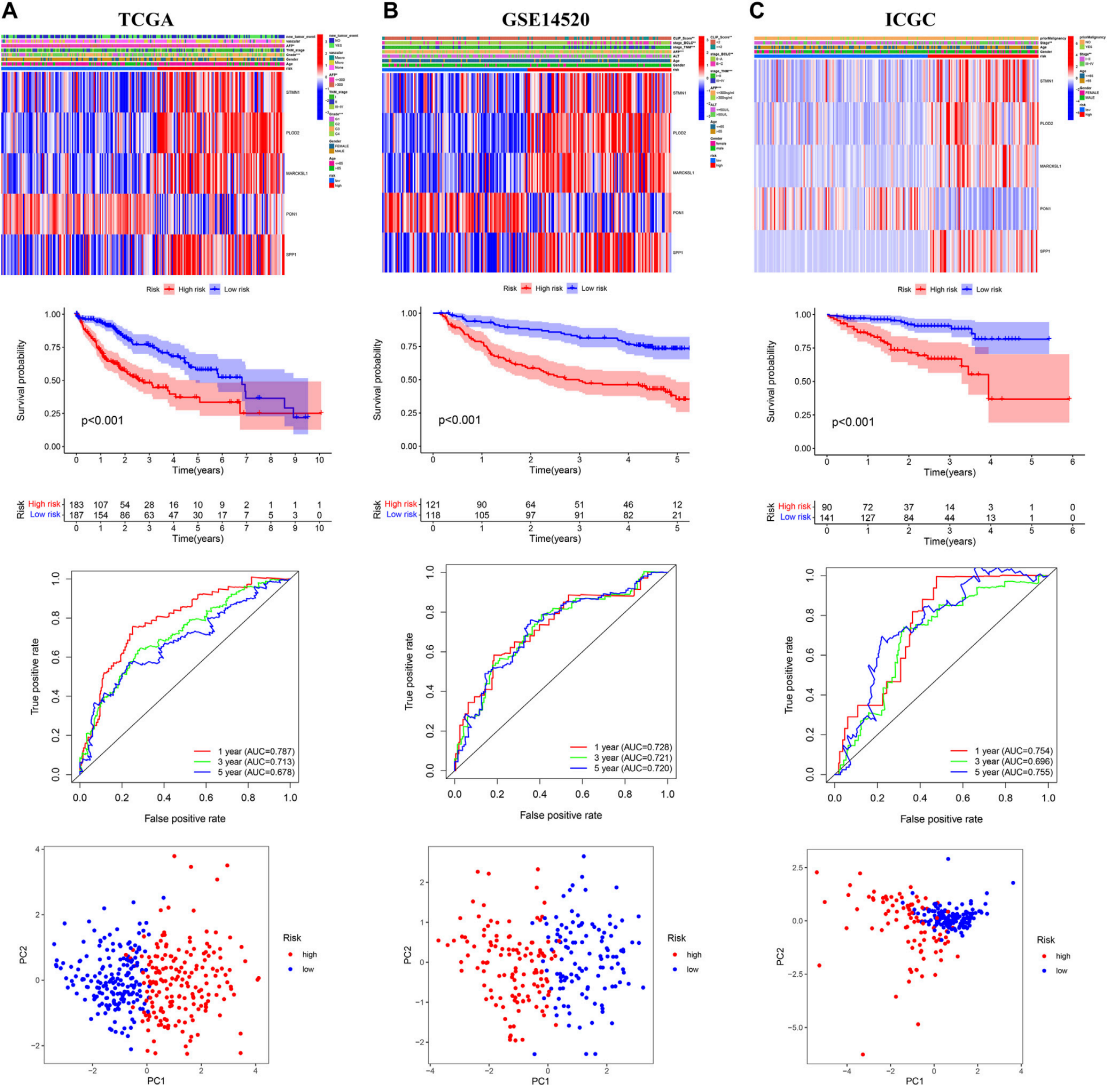
Internal and external validation of the prognostic model in the three independent cohorts **(A)** TCGA **(B)** GSE14520 **(C)** ICGC.

The OS of high-risk patients were significantly reduced relative to the low-risk patients in internal (TCGA, *n* = 370; GSE14520, *n* = 239) and external (ICGC, *n* = 231; GSE116174, *n* = 64) validation cohorts (Figures 3A–C; Supplementary Figure S4A). In the TCGA cohort, the AUC values of 1-year, 3-year, and 5-year OS predicted by risk score were 0.787, 0.713, and 0.678 respectively (Figure 3A); In the GSE14520 cohort, the AUC values of 1-year, 3-year, and 5-year OS predicted by risk score were 0.728, 0.721, and 0.720 respectively (Figure 3B); In the ICGC cohort, the AUC values of 1-year, 3-year and 5-year OS predicted by risk score were 0.754, 0.696, and 0.755 respectively (Figure 3C). In the GSE116174 cohort, the AUC values of 1-year, 3-year, and 5-year OS predicted by risk score were 0.751, 0.610, and 0.723 respectively (Supplementary Figure S4B). The principal component analysis (PCA) showed satisfactory separation between the high- and low-risk groups (Figure 3C). Among the five genes, only *PON1* was upregulated in the low-risk group, and the other four genes were upregulated in the high-risk group (Figures 3A–C; Supplementary Figure S4C).

### Clinical Correlation Analysis and Subgroup Survival Analysis

**FIGURE 4:**
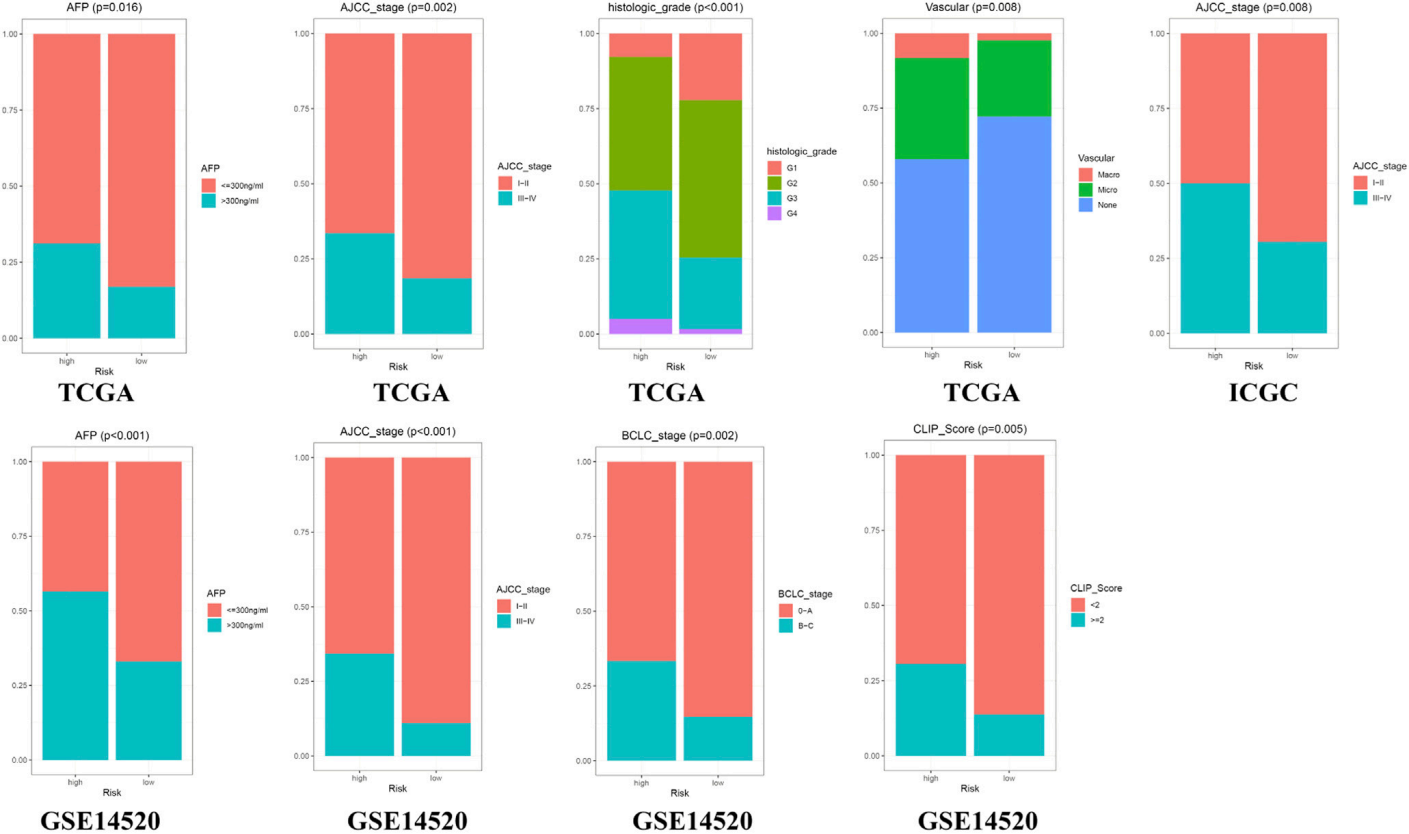
Clinical correlation analysis in the three independent cohorts.

**FIGURE 5:**
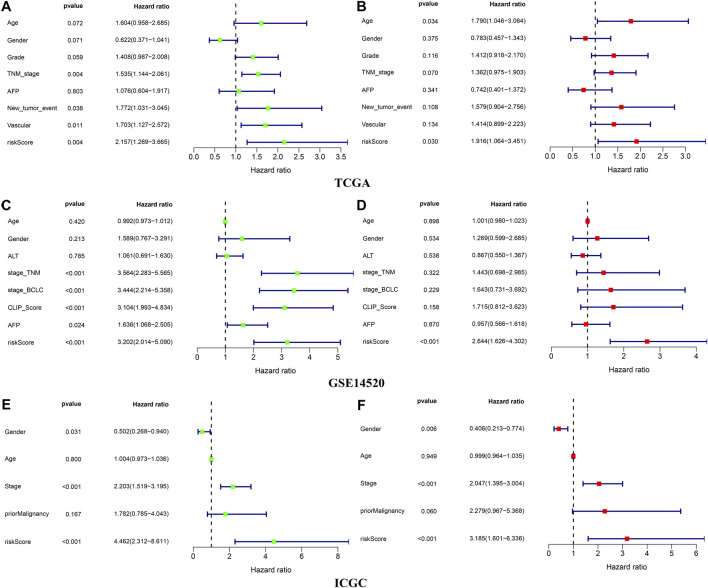
Independence validation of the risk score in the three independent cohorts **(A,B)** TCGA **(C,D)** GSE14520 **(E,F)** ICGC. *green represent univariate Cox analysis, red represent multivariate Cox analysis.

**FIGURE 6:**
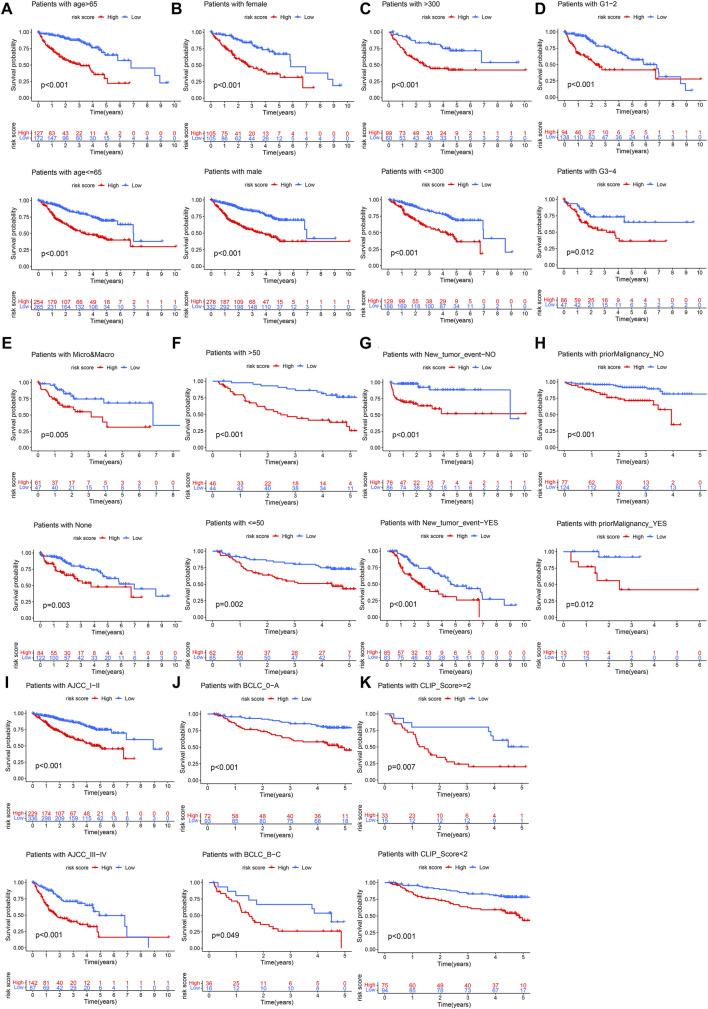
Clinical subgroup survival analysis. **(A)** Age, **(B)** Gender, **(C)** AFP, **(D)** Histology grade, **(E)** Vascular tumor cell type, **(F)** ALT, **(G)** New tumor event after initiative treatment, **(H)** Prior malignancy, **(I)** AJCC stage, **(J)** BCLC stage, **(K)** CLIP score.

The Chi-square test showed that the patients with AFP >300 ng/ml, tumor poor differentiation (grade 3–4), micro and macro vascular tumor invasion, advanced stage (AJCC III-IV, BCLC stage B-C, and CLIP score>2) exhibited a higher risk score (Figure 4). The independent-samples t-tests suggested that the risk score distributed significantly differently in patients with different AFP levels, histology grades, vascular tumor cell type, AJCC stage, BCLC stage, and CLIP score (Supplementary Figure S5). The univariate and multivariate Cox regression analysis demonstrated that the risk score was an independent risk factor for the OS of patients in the three independent cohorts (Figures 5A–C; Supplementary Figures S4D,E). In 22 subgroups assigned by clinical characteristics, the OS of high-risk patients were significantly reduced compared to the low-risk patients in each subgroup (*p* < 0.05, Figures 6A–K).

### Uncovering the Potential Molecular Mechanism of the Prognostic Signature

**FIGURE 7:**
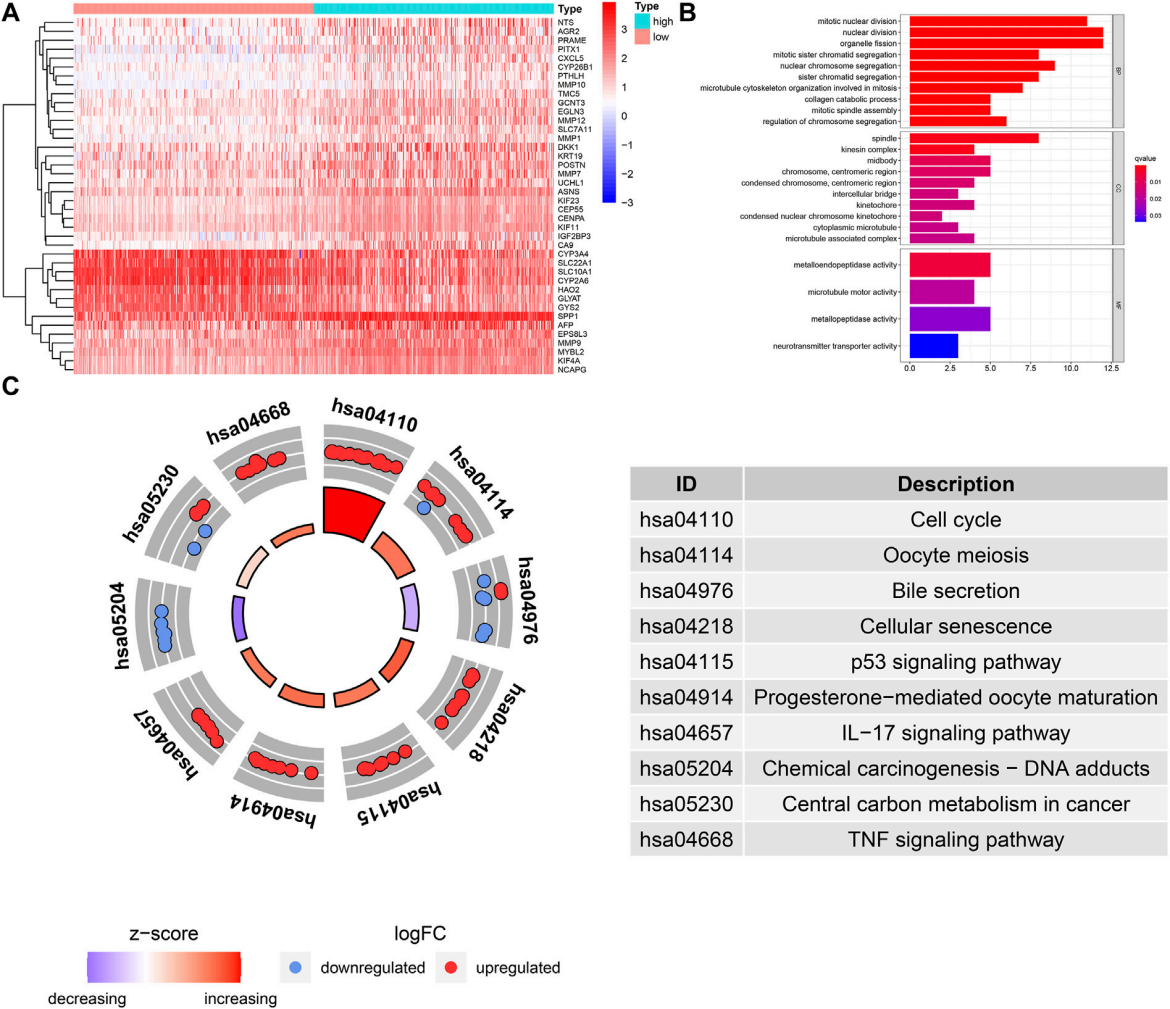
GO and KEGG functional enrichment analysis for the DEGs between different risk groups. **(A)** The heatmap of DEGs between different risk groups. **(B)** GO term annotation. **(C)** KEGG pathway enrichment analysis.

We identified the DEGs between high- and low-risk groups (Figure 7A), GO annotation found that these DEGs were involved in nuclear division, mitotic nuclear division, and organelle fission, etc. (Figure 7B). KEGG enrichment analysis showed that cellular senescence, cell cycle, p53 signaling, TNF signaling-related pathways were upregulated in the high-risk group, and it is worth mentioning that the genes with a positive risk coefficient were also involved in the pathways which were significantly upregulated in the high-risk group. While chemical carcinogenesis and bile secretion related pathways were upregulated in the low-risk group (Figure 7C).

### Comparison of the Immune Infiltration in Different Risk Groups

**FIGURE 8:**
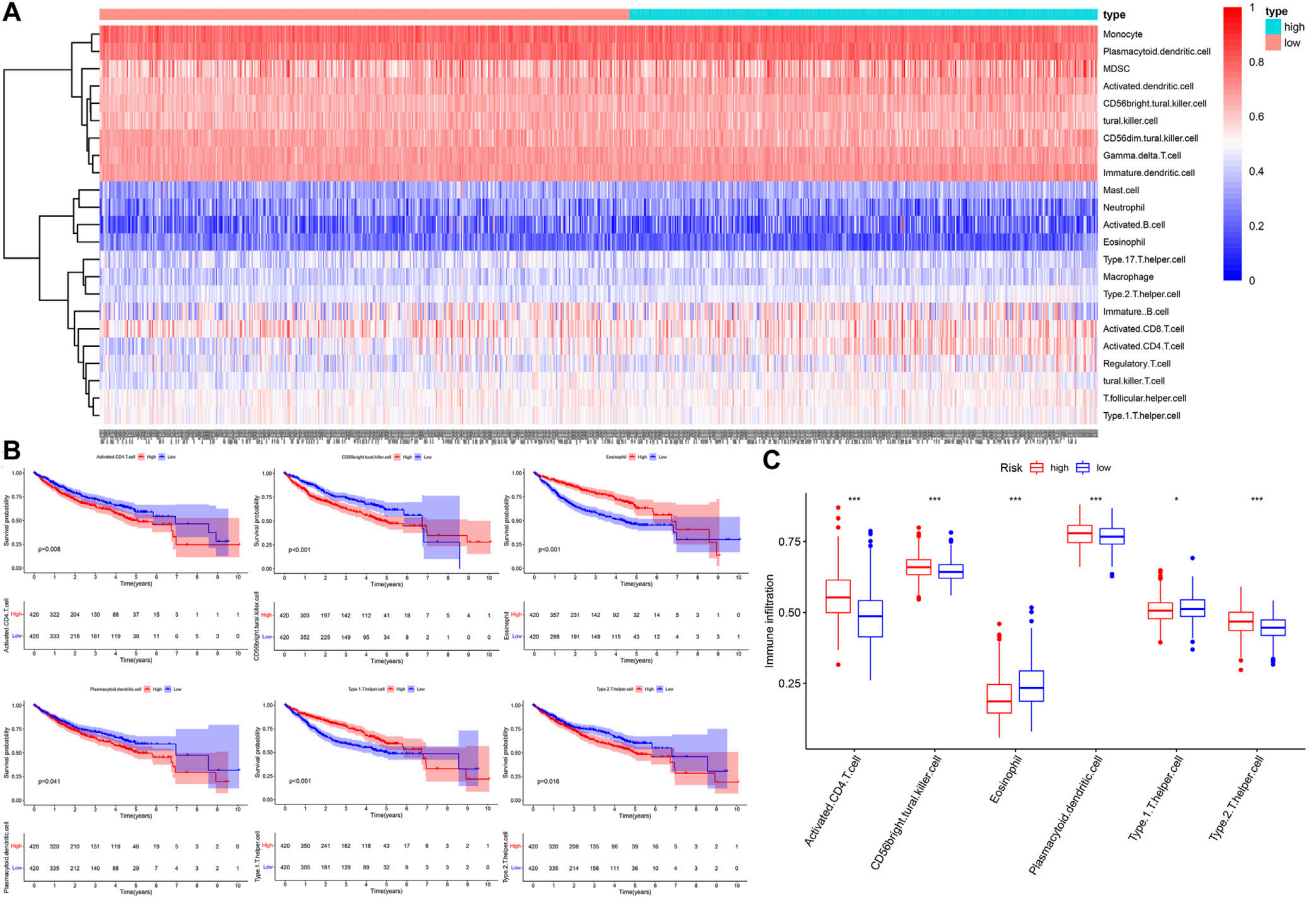
The landscape of immune cells infiltration. **(A)** The heatmap of 23 types of immune cells infiltration. **(B)** The Kaplan–Meier survival analysis for immune cells infiltration. **(C)** The boxplot of immune cells infiltration between different risk groups.

We performed quantification of 23 types of immune cells infiltration abundance via the ssGSEA algorithm (Figure 8A). Based on the median NES, the patients were divided high- and low- infiltration groups. The Kaplan-Meier survival curves showed that the patients with higher infiltration levels of activated CD4 T cell, CD56 bright natural killer cell, plasmacytoid dendritic cell, and type 2 T helper cells had poor prognosis (*p* < 0.05, Figure 8B), and these four kinds of immune cells exhibited a higher infiltration level in the high-risk group (Figure 8C). The patients with higher infiltration levels of eosinophil and type 1 T helper cells showed better prognosis (*p* < 0.05, Figure 8B), and these two kinds of immune cells exhibited a higher infiltration level in the low-risk group (Figure 8C).

### Investigation Treatment Strategy for Different Risk Groups

**FIGURE 9:**
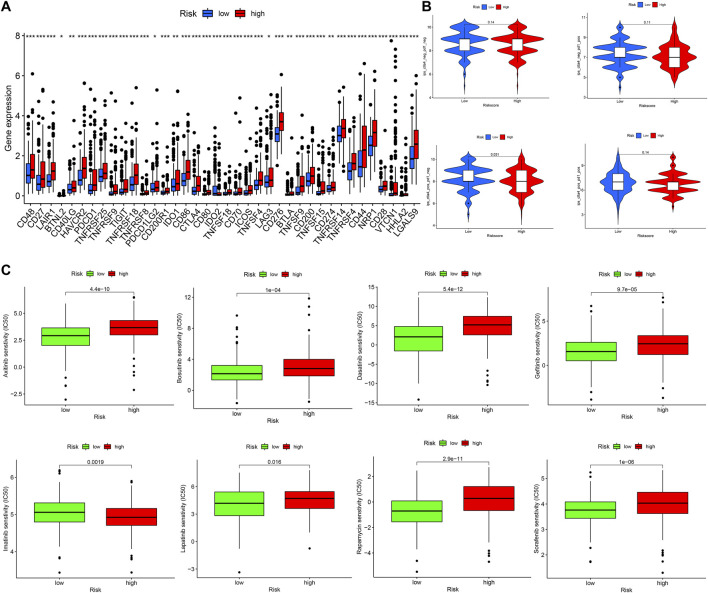
Investigation treatment strategy for different risk groups **(A)** The boxplot of immune checkpoints in different risk groups **(B)** The IPS of ICIs in different risk groups **(C)** The IC50 of anticancer drugs in different risk groups.

First, we compared the expression level of immune checkpoints between the high-and low-risk groups, the expression level of *TIGIT, CTLA4, PDCD1, LAG3,* and *CD274(PDL1)*, etc., were all significantly upregulated in the high-risk group (Figure 9A). Next, we obtained the immunophenoscore (IPS) of patients in the TCGA cohort from The Cancer Immunome Atlas (https://tcia.at/home) (Charoentong et al., 2017). In general, the higher the IPS, the more sensitivity to immune checkpoint inhibitors (ICIs); the IPS of CTLA4 (+) and PD1 (−) in the low-risk group was higher than that of the high-risk group with statistical significance (Figure 9B), indicating that the low-risk group was more likely to benefit from immunotherapy with anti-CTLA4 ICIs. In addition, we also compared the difference of anticancer drug sensitivity between the high- and low-risk groups by calculating the IC50 of anticancer drugs with the R package pRRophetic. The IC50 of sorafenib, rapamycin, lapatinib, and gefitinib, etc. in the low-risk group was significantly lower than that of the high-risk group, but the IC50 of imatinib in the high-risk group was lower than that of the low-risk group with statistical significance (Figure 9C).

## Discussion

Hepatocellular carcinoma (HCC) is a common malignant tumor, and its etiology and pathogenesis have not been fully elucidated (Yang et al., 2019). It is closely related to hepatitis virus infection, aflatoxin, and bile acid to varying degrees (Fattovich et al., 2004), these factors could cause DNA damage in hepatocytes and then trigger a series of cellular reactions, mainly including damage signal transduction and apoptosis induced by DNA repair (Tanaka et al., 2008). If DNA damage could not be repaired correctly and accumulated continuously, it could lead to malignant transformation of hepatocytes and eventually lead to HCC (Gillman et al., 2021). Therefore, DNA damage and repair (DDR) is an important molecular mechanism for the occurrence and development of HCC, and further study of it will lay a foundation for the comprehensive treatment of HCC.

In our research, we found that the HCC patients with different DDR molecular subtypes had different clinical outcomes. C1 was defined as DDR active subtype, and its prognosis was significantly worse than C2, which confirmed that the activity of DDR was indeed related to the progression of HCC. Considering the complex correlation between DDR and genomic instability (Kerzendorfer and O’Driscoll, 2009), we identified the DEGs between C1 and C2 further. The results showed that 193 of 239 DEGs were significantly correlated with the OS of HCC (*p* < 0.001), therefore, the difference in the expression of these DEGs may be the potential cause of the difference in prognosis between C1 and C2. After lasso and multivariate Cox regression analysis, a risk score consisting of five genes was established in the training cohort. The training cohort was a cohort with a large sample size (*n* = 609) merged by TCGA and GSE14520 datasets. In order to test the reliability of the risk score, we carried out internal and external validation. The AUC values for the risk score to predict 1, 3, and 5 year OS were all greater than 0.6 in the four independent cohorts, indicating that the risk score had high accuracy in the OS prediction of HCC. The risk score could be regarded as an independent prognosis indicator as shown by univariate and multivariate Cox regression analysis. The patients were divided into 22 subgroups according to clinical characteristics, the high-risk patients’ OS decreased obviously in each subgroup, which proved that the risk score was applicable to patients with different clinical characteristics.

To clarify the reasons for the difference in OS between high- and low-risk groups, we explored it from three aspects: clinicopathological features, molecular mechanism, and immune infiltration. First, from the perspective of clinical relevance, we found the presentation of risk score was higher in patients with AFP >300 ng/ml, tumor poor differentiation (grade 3–4), vascular micro and macro invasion, advanced stage (AJCC III-IV, BCLC stage B-C, and CLIP score >2), manifested that the higher risk score represented rapid tumor progression and stronger invasiveness. Secondly, from the perspective of molecular mechanism, we found that the cell cycle, p53 signaling, TNF signaling related pathways were positively enriched in the high-risk group, while chemical carcinogenesis, bile secretion related pathways were upregulated in the low-risk group, these results suggested that the risk score may affect tumor progression by regulating tumor cell proliferation and metabolism. Interestingly, the expression of genes involved in cellular senescence was up-regulated in the high-risk groups, indicating that the aging tumor microenvironment may have an adverse impact on the prognosis of HCC and played a critical role in the malignant progression of HCC. Thirdly, in terms of immune infiltration, we found that the infiltration level of six kinds of immune cells had a significant impact on the OS of HCC. The patients with higher infiltration abundance of activated CD4 T cell, CD56 bright natural killer cell, plasmacytoid dendritic cell (pDC), and type 2 T helper (Th2) cells had adverse prognosis, and these four kinds of immune cells exhibited a higher infiltration level in the high-risk group. The patients with higher infiltration levels of eosinophil and type 1 T helper (Th1) cells showed a favorable prognosis, and these two kinds of immune cells exhibited a lower infiltration level in the high-risk group. Previous reports have pointed out that activated CD4 T cells have the ability to inhibit tumor, it could not only directly produce toxic effects on tumor cells, but also play an auxiliary role in the activation and proliferation of CD8 T cells (Toes et al., 1999; Gerloni and Zanetti, 2005). However, we found that the activated CD4 T cells showed higher infiltration in the high-risk group and were correlated with unfavorable survival outcomes. In the tumor microenvironment, pDC could not effectively activate T cells to kill tumor cells, but induced the production of various regulatory T cells (Tregs) and promoted the immune escape of tumor cells (Conrad and Gilliet, 2013). Th1 was considered to be the most important helper cell type in tumor immunity, it can directly kill tumor cells by releasing cytokines that activate death receptors on the surface of tumor cells (LaCasse et al., 2011). Th2 mediated immunity was traditionally considered to be conducive to tumor growth, which can not only promote angiogenesis but also inhibit cell-mediated immunity and subsequent tumor cell killing (Ellyard et al., 2007). Therefore, the decreased antitumor immune response may be the potential reason that resulted in the poor prognosis of the high-risk group.

Up to now, the five genes have attracted extensive attention in the field of cancer. Zhang (Zhang et al., 2020) found that the upregulation of *STMN1* promoted the growth of HCC by triggering the MET pathway. Aronova (Aronova et al., 2018) found that *STMN1* was overexpressed in adrenocortical carcinoma and promoted a more invasive phenotype *in vitro*. Jiang (Jiang et al., 2018) found that *STMN1* promotes the proliferation, migration, and invasion of esophageal squamous cell carcinoma by activating the PI3K pathway. He (He et al., 2016) found that *STMN1* promotes the growth and invasion of endometrial carcinoma by mediating the secretion and activation of *MMP2* and *MMP9* proteins. Li (Li et al., 2015) found that overexpression of *STMN1* was related to the proliferation, migration, invasion, and apoptosis of human skin squamous cell carcinoma. Bao (Bao et al., 2017) found that the increased expression of *STMN1* is associated with the progression and chemoresistance of lung squamous cell carcinoma. Bai (Bai et al., 2017) found that the high level of *STMN1* in patients with gastric cancer was related to chemoresistance and poor prognosis. Shu (Shu et al., 2017) and Ding (Ding et al., 2020) found that *PON1* had an important diagnostic reference value for AFP negative HCC and was helpful to predict the microvascular invasion of HCC. Yu (Yu et al., 2018) found that the decreased expression of *PON1* represented the high invasiveness of HCC and was closely related to the recurrence and metastasis of HCC. Cao (Cao et al., 2021) found that variations in *PON1* glycosylation may help to distinguish AFP negative HCC from cirrhosis. *PLOD2* has been regarded as an oncogene and its upregulation is closely associated with malignant behavior and poor prognosis in multiple cancers (Du et al., 2017b). For example, Du (Du et al., 2020) found that *PLOD2* promotes aerobic glycolysis and cell progression of colorectal cancer by up regulating *HK2*; Wan (Wan et al., 2020) found that *PLOD2* regulates the migration, invasion and EMT of endometrial cancer cells through *PI3K/Akt* signaling pathway; Kiyozumi (Kiyozumi et al., 2018) found that under hypoxia, *PLOD2* promoted the invasion and migration of gastric cancer cells and led to peritoneal dissemination of gastric cancer; Sheng (Sheng et al., 2019) found that *PLOD2* promotes drug resistance in laryngeal cancer by promoting tumor stem cell like characteristics; Okumura (Okumura et al., 2018) found that hypoxia induced *PLOD2* is a key regulator of epithelial mesenchymal transformation and chemotherapy tolerance in biliary cancer; Du (Du et al., 2017a) found that *PLOD2* is regulated by PI3K/akt-foxa1 axis and promotes the metastasis of non-small cell lung cancer; Noda (Noda et al., 2012) found that *PLOD2* expression was significantly correlated with tumor size and visible intrahepatic metastasis of HCC, which was an independent risk factor for poor prognosis. Liang (Liang et al., 2020) found that *MARCKSL1* promotes the progression of lung adenocarcinoma by regulating epithelial-mesenchymal transition. Zhang (Zhang et al., 2017) found that *SPP1* promotes immune escape of lung adenocarcinoma by mediating macrophage polarization. Zeng (Zeng et al., 2018) found that *SPP1* promotes ovarian cancer progression via Integrin β1/FAK/AKT signaling pathway. Wang (Wang et al., 2019) found that *SPP1* can promote cell growth in miR-181c targeted HCC.

At present, tumor treatment has stepped into the era of precision medicine, that is, an emerging medical model based on individualized medicine and integrating gene detection, biological information, and big data science. As an important part of establishing a clinical decision-making system, tumor prognosis evaluation has become an important research content of precision medicine. Considering the prognosis of HCC is still a great challenge for medicine in its current stage, the five-gene risk score proposed in the research may provided new insights into the individualized evaluation of HCC prognosis. However, there were still some limitations that should be acknowledged in our work, the results were based on the public data that we did not validate in our samples, and the work was conducted without an experimental mechanism study.

## Conclusion

The five-gene risk score proposed in the research may provide new insights into the individualized evaluation of HCC prognosis.

## Data Availability

Publicly available datasets were analyzed in this study. This data can be found here: The datasets analyzed for this study were obtained from The Cancer Genome Atlas (TCGA, https://portal.gdc.cancer.gov/), International Cancer Genome Consortium database (ICGC, https://dcc.icgc.org/releases/current/Projects/LIRI-JP), Gene Expression Omnibus(GEO, https://www.ncbi.nlm.nih.gov/geo/), and the molecular signatures database (MSigDB, http://www.gsea-msigdb.org/gsea).

## Abbreviations

HCC, hepatocellular carcinoma; DDR, DNA damage repair; TCGA, the cancer genome atlas; ICGC, international cancer genome consortium; GEO, gene expression omnibus; PRGs, prognostic related genes; DEGs, differential expressed genes; GO, gene ontology; KEGG, kyoto encyclopedia of genes and genomes; KM, Kaplan-Meier; LASSO, least absolute shrinkage and selection operator; ROC, receiver operating characteristic; AUC, area under curve; OS, overall survival; NES, normalized enrichment score; GSEA, gene set enrichment analysis; ssGSEA, single sample gene set enrichment analysis; IPS, immunophenoscore; ICIs, immune checkpoint inhibitors.
