# Supplementary figures and images for: A Five-Gene Signature Associated With DNA Damage Repair Molecular Subtype Predict Overall Survival for Hepatocellular Carcinoma
Source: Front Genet. 2022 Jan 20;13:771819. doi: 10.3389/fgene.2022.771819 (PMC8811360; doi:10.3389/fgene.2022.771819)

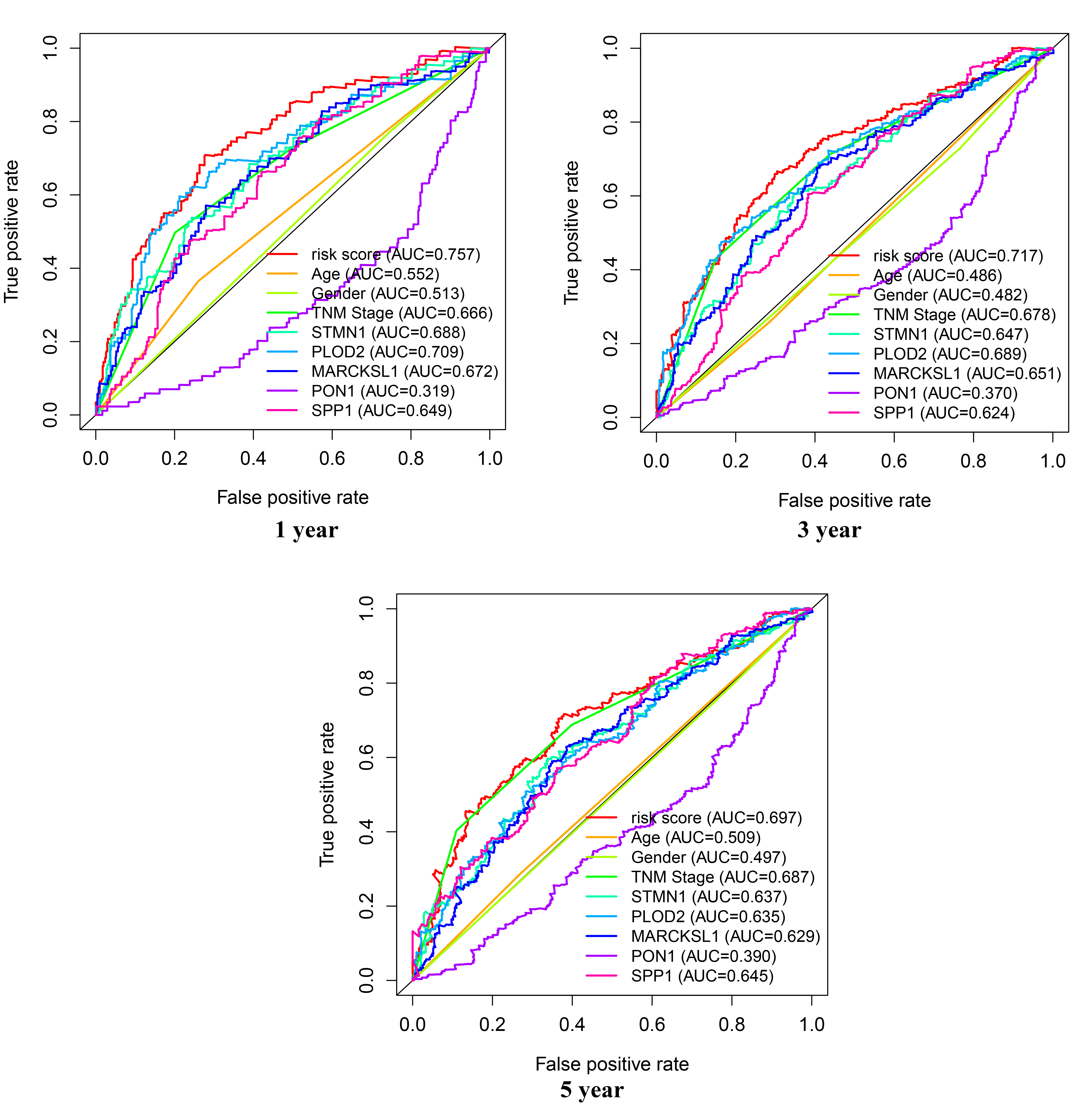

Supplement: Supplementary file 5 [file Image2.TIF]

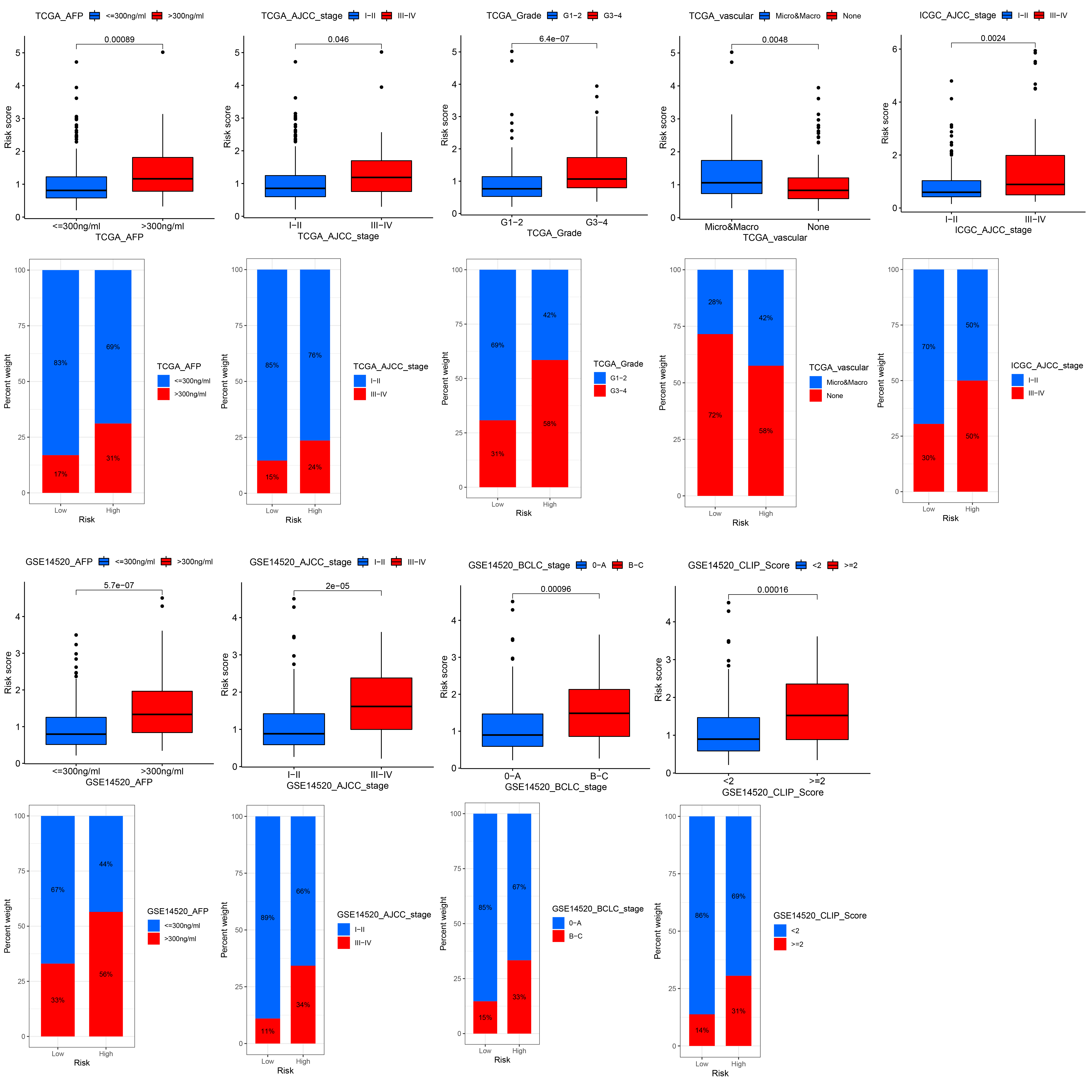

Supplement: Supplementary file 8 [file Image5.TIF]
