# Supplementary material for: A Five-Gene Signature Associated With DNA Damage Repair Molecular Subtype Predict Overall Survival for Hepatocellular Carcinoma
Source: Front Genet. 2022 Jan 20;13:771819. doi: 10.3389/fgene.2022.771819 (PMC8811360; doi:10.3389/fgene.2022.771819)
Supplement: Supplementary file 7 [file Table3.DOCX]

Table-1 The clinicopathological information for all cohorts

|  | TCGA | GSE14520 | ICGC | GSE116174 |  |  |  |  |  |
| --- | --- | --- | --- | --- | --- | --- | --- | --- | --- |
| Survival status |  | | | |  |  |  |  |  |
| alive | 240 | 143 | 189 | 37 |  |  |  |  |  |
| dead | 130 | 96 | 42 | 27 |  |  |  |  |  |
| Gender |  | | | |  |  |  |  |  |
| male | 249 | 189 | 170 | 6 |  |  |  |  |  |
| female | 121 | 28 | 61 | 58 |  |  |  |  |  |
| Age |  | | | |  |  |  |  |  |
| <=65 | 232 | 198 | 89 | 55 |  |  |  |  |  |
| >65 | 138 | 19 | 142 | 9 |  |  |  |  |  |
| HBV |  | | | |  |  |  |  |  |
| positive |  | | | 47 |  |  |  |  |  |
| negative |  | | | 17 |  |  |  |  |  |
| alcohol |  | | | |  |  |  |  |  |
| yes |  | | | 13 |  |  |  |  |  |
| none |  | | | 51 |  |  |  |  |  |
| Grade |  | | | |  |  |  |  |  |
| G1 | 55 |  | | |  |  |  |  |  |
| G2 | 177 |  | | |  |  |  |  |  |
| G3 | 121 |  | | |  |  |  |  |  |
| G4 | 12 |  | | |  |  |  |  |  |
| AJCC TNM stage |  | | | |  |  |  |  |  |
| I&II | 256 | 168 | 141 | 53 |  |  |  |  |  |
| III&IV | 90 | 49 | 90 | 11 |  |  |  |  |  |
| priorMalignancy |  | | | |  |  |  |  |  |
| none |  | | 201 |  |  |  |  |  |  |
| yes |  | | 30 |  |  |  |  |  |  |
| AFP |  | | | |  |  |  |  |  |
| <=300ng/ml | 197 | 120 |  | |  |  |  |  |  |
| >300ng/ml | 62 | 97 |  | |  |  |  |  |  |
| vascular_tumor_cell_type | |  | | |  |  |  |  |  |
| none | 206 |  | | 35 |  |  |  |  |  |
| micro&macro | 108 |  | | 29 |  |  |  |  |  |
| new_tumor_event_after_initial_treatment | | |  | |  |  |  |  |  |
| none | 162 |  | | |  |  |  |  |  |
| yes | 168 |  | | |  |  |  |  |  |
| BCLC stage |  | | | |  |  |  |  |  |
| 0-A |  | 165 |  | |  |  |  |  |  |
| B-C |  | 52 |  | |  |  |  |  |  |
| CLIP_Score |  | | | |  |  |  |  |  |
| <2 |  | 169 |  | |  |  |  |  |  |
| >=2 |  | 48 |  | |  |  |  |  |  |
| ALT |  | | | |  |  |  |  |  |
| <=50U/L |  | 127 |  | |  |  |  |  |  |
| >50U/L |  | 90 |  | |  |  |  |  |  |
